# HPV testing for cervical cancer screening: technical improvement of laboratory logistics and good clinical performance of the cobas 6800 in comparison to the 4800 system

**DOI:** 10.1186/s12905-019-0743-0

**Published:** 2019-03-25

**Authors:** Helena Frayle, Silvia Gori, Martina Rizzi, Bianca Nives Graziani, Elisa Vian, Paolo Giorgi Rossi, Annarosa Del Mistro

**Affiliations:** 1 Immunology and Molecular Diagnostic Oncology Unit, Veneto Institute of Oncology IOV-IRCCS, Via Gattamelata, 64, 35128 Padova, Italy; 2 Pathology Unit, Ospedale di Santorso, Via Garziere, 42-36014 Santorso (VI), Italy; 3 Microbiology and Virology Unit, Clinical Pathology Department, Ospedale Ca’ Foncello, Piazza Ospedale, 1-Treviso, Italy; 4 Epidemiology Unit, AUSL Reggio Emilia, IRCCS, Reggio Emilia, Italy

**Keywords:** Cervical cancer screening, HPV testing, Automation, Technical improvement, Clinical performance

## Abstract

**Background:**

European guidelines for cervical cancer screening now recommend the use of clinically validated assays for high-risk HPV-DNA sequences as primary test in women older than 30 years, performed in centralized laboratories, and run on systems providing automated solutions for all steps.

**Methods:**

We conducted a comparison study, according to the international guidelines, nested within the organized population-based cervical screening program, between the cobas 4800 and 6800 systems (Roche Diagnostics), to evaluate accuracy and reproducibility of HPV test results and laboratory workflow. In Italy implementation of HPV cervical screening is under way on a regional basis; in Veneto it started in June 2015, following a piloting phase; the assay in use in the three centralized laboratories is the cobas 4800 HPV test, run on the cobas 4800 system. Comparison of HPV results with a new version of the assay (cobas 6800/8800 HPV) run on the cobas 6800 system, and intra- and inter-reproducibility analyses have been conducted in samples collected in PreservCyt medium (Hologic) from women without and with a subsequent diagnosis of high-grade lesion.

**Results:**

Samples from women older than 30 years attending organized cervical cancer screening were used. Clinical sensitivity and specificity were evaluated on 60 cases and 925 controls, respectively; intra-laboratory reproducibility and inter-laboratory agreement by the 6800 system were evaluated on 593 and 460 specimens, respectively. Our results showed a very high agreement (> 98%) for overall qualitative results between the two systems; clinical sensitivity and specificity of the HPV assay run on 6800 were non-inferior to those of the HPV assay run on 4800 (*p* = 0,0157 and *p* = 0,0056, respectively, at the recommended thresholds of 90 and 98%); kappa values of 0.967 and 0.969 were obtained for intra- and inter-laboratory reproducibility analyses in the 6800 system. The 6800 platform displayed several technological improvements over the 4800 system, with higher throughput and laboratory productivity, and lower operator’s hands-on time.

**Conclusions:**

The new cobas 6800/8800 HPV assay run on the 6800 instrument is suitable for use in large centralized laboratories included within population-based cervical cancer screening programs.

## Background

DNA testing for HPV oncogenic types [1] as primary test in organized cervical cancer screening is more effective than cytology in women above 30 years of age [2]. European [3] and Italian [4] guidelines recommend the use of clinically validated HPV assays [5], to be performed in centralized laboratories.

In order to allow testing in large centralized laboratories and to ensure diagnostic accuracy, the assays must be run on systems providing automated solutions for both pre-analytical and analytical phases, starting from specimen primary tubes. Operator interaction, test throughput, workflow and system maintenance requirements are important determinants and substantial differences among some available automated systems have been shown [6]; for most of the validated assays, at present, two or more diagnostic systems are necessary to guarantee the requested workload of a centralized laboratory. Technological improvements of the instrumentations in use with some of the validated assays have been released and/or are on the way; their clinical performance, consistency of results and operational characteristics are best evaluated by real-world studies.

In Italy, the Ministry of Health has introduced the implementation of HPV testing for cervical screening in the National Preventive Plan 2014–2018; cancer screenings are managed at regional level, and all regions are expected to comply by the year 2018. In the Veneto region, the use of DNA HPV testing in primary cervical screening was piloted in five organized programs from April 2009 to May 2015 [7–9], and fully implemented in all programs since June 2015. The HPV assay actually in use is the clinically validated cobas 4800 HPV test (Roche Diagnostics) [10, 11], run on the cobas 4800 system. More recently, a new version of the assay (cobas 6800/8800 HPV) for use on the cobas 6800 and cobas 8800 systems [12] has been developed and CE/IVD (Conformité Européenne/in vitro diagnostics) labeled.

The aim of our study is the evaluation of the cobas HPV assay performed on the new 6800 platform in comparison to the 4800 for use in cervical cancer screening.

## Methods

### Aim, design and setting of the study

The study was nested within the organized population-based cervical cancer screening, and carried out in one of the three centralized HPV laboratories of the Veneto region (Italy), serving five programs. The HPV screening protocol is applied to women older than 30 years and includes HPV testing and cytology triage of HPV-positive samples, followed by immediate colposcopy in case of ASC-US (Atypical Squamous Cells of Undetermined Significance) or more severe diagnosis, or HPV retesting at 1-yr recall in case of negative cytology [4]. Women testing HPV-negative will be re-invited at 5-yrs interval.

The study design aimed at evaluating clinical sensitivity and specificity and assay reproducibility by the cobas 6800 HPV assay in comparison to the cobas 4800 assay, according to the Meijer’s criteria [5], as well as technical performance and laboratory workflow of the two platforms.

The instrumentation for the study (p 480 v2 and cobas 6800) was temporarily provided by Roche Diagnostics. The inter-laboratory analyses were performed at the Microbiology and Virology Unit of the General Hospital of Treviso, where a cobas 6800 system is in use for other assays, and the HPV dedicated software was implemented.

### Clinical samples and HPV testing by cobas 4800

All samples were previously tested by the cobas 4800 HPV assay, based on real-time polymerase chain reaction (PCR) technology, by use of the 4800 system, that provides full sample preparation (cobas × 480) and HPV detection (cobas z 480 analyzer); the two instruments are physically separated and require the manual transfer of the reaction plate. The method detects 14 HPV types (the 12 designed as high-risk by the IARC, plus types 68 and 66), provides individual HPV genotype results for HPV16 and HPV18, while detecting the other 12 types as a pool (other HR), and includes an internal quality control (beta-globin) for each sample (in the screening report only the result for all hrHPV types as a pool is included). The primary vial is mixed and decapped/recapped by the p480 v1 instrument.

Residual material of cervical samples collected in PreservCyt medium (Hologic, Bedford, MA, USA) from women older than 30 years, attending cervical cancer screening was used. Consecutive specimens were collected during February 1 through May 31, 2017 and during December 1, 2017 through February 15, 2018, and stored for no longer than 6 months before testing by 6800; selected samples were also included, in order to increase the number of cases (25 samples obtained from women with high-grade lesions and stored for up to 3 years at 4 °C before testing for the present study) and of HPV-positive samples (to provide a 25–30% prevalence of high-risk HPV for the reproducibility analyses). The 25 samples stored longer than 6 months were re-tested by cobas 4800 to verify amplificability and consistency of results; all samples gave a valid result (1 was invalid only for the HPV channels previously negative) with overlapping qualitative results and Ct (cycle threshold) values, and were deemed suitable for inclusion in the study.

For ethical reasons, all samples were anonymized before HPV analysis. The study has been approved by the local Ethical Committee (EC code 2017–07 plus EM 193/2017).

### Sample processing and cobas 6800 HPV assay

Primary vials were initially processed by the p 480 v2 (with software 2.1.1) instrument, that performed spin-mixing, cap removal, barcode alignment and transfer of 2-ml sample aliquots to secondary vials, and recapping with the original primary vial cap.

The secondary vials were then manually transferred to the cobas 6800 system, a fully automated unit that provides full sample preparation and HPV detection without further intervention by the operator.

Both cobas HPV real-time PCR assays detect the same 14 types of HPV, use the same primers and probes, and provide partial genotyping for HPV16 and HPV18. The two assays do however differ in their Thermal Cycling (CT) profile (the cobas 6800/8800 runs a universal thermal cycling profile to allow for mixed batching of different PCR tests), as well as in the elution sample volume amplified (50 μl on 6800/8800 vs 150 μl on 4800 out of a 400 μl aliquot of extracted nucleic acids). Both HPV assays were performed according to the manufacturer’s instructions. Each plate, besides the 2 assay’s controls, contained 92 clinical specimens and 2 additional samples (selected from 10 internal and 5 external quality controls, and 2 clinical samples previously found to be invalid by the cobas 4800 HPV test).

### Statistical analyses

Clinical sensitivity and clinical specificity were evaluated on samples from women with a histologically confirmed high-grade lesion (CIN2+) and on samples from women with no or low-grade lesions, respectively; the results with the 6800 assay were compared to those with the 4800 by a non-inferiority score test, according to the thresholds (relative sensitivity of at least 90% and relative specificity > 98%) recommended by Meijer et al. [5]. The HPV results obtained by the two systems were assessed using overall percentage agreement of qualitative (positive/negative) results and type (HPV16, HPV18, other HPV) agreement; complete and partial type concordance were evaluated also by hierarchical categorization (i.e., HPV16 alone and with any other type; HPV18 alone and with any non-16 other type; non-16/non-18 other HPV types only). For discordant results, the cycle threshold (Ct) values were also analyzed. The intra-laboratory reproducibility and the inter-laboratory agreement were evaluated by calculating the kappa values and 95% confidence intervals (95%CI) by bootstrap analysis (replications = 1000; bias corrected); we also report the squared correlation coefficient R^2^ (variation in Y explained by X/variation in Y) between the Ct values.

## Results

The cases’ group comprised 62 samples from women (mean age 41 yrs.; median age 40 yrs.; range 30–59 yrs) with a histologically confirmed high-grade lesion (32 CIN2, 27 CIN3, 2 squamous cell carcinomas, 1 adenocarcinoma) diagnosed either at baseline (42 with HPV-positive/cytology positive results, and 3 with persistent HPV positivity at 1-yr recall) or during follow-up (17 cases). The controls’ group comprised 925 women (mean age 46 yrs.; median age 45 yrs.; range 30–68 yrs) with no or low-grade lesions. By the cobas 4800 HPV assay, all the cases and 67/925 (7.2%) controls were HPV-positive.

A valid cobas 6800 HPV test result has been obtained for all but two samples (both from cases, comprising the partially invalid at 4800 re-testing, excluded from the study); 59/60 (98.4%) cases and 73/925 (7.9%) controls gave a positive result. A concordant result was recorded in 59/60 (98.4%) cases and in 915/925 (98.9%) controls. In Table 1 the qualitative results (positive/negative), as well as the HPV type distribution by hierarchical categorization, of all the samples are reported.

**Table 1:** Comparison of HPV test results on cobas 4800 and cobas 6800 systems on samples from women with no or low grade lesions (controls, *N* = 925) and women with CIN2+ lesions (cases, *N* = 60); HPV prevalence by the cobas 4800 assay was 7.2% among controls and 100% among cases

Cobas 4800 HPV test results	Cobas 6800 HPV test results				Total
		Positive			
	Negative	HPV16^a^	HPV18^b^	Other HPV	
CONTROLS (<CIN 2)					
Negative	850	4	3	1	858
Positive					
HPV16^a^		13			13
HPV18^a,b^			5		5
Other HPV	2	2	4	41	49
Total	852	19	12	42	925
CASES (CIN 2+)					
Positive					
HPV16^a^		28			28
HPV18^a,b^			4		4
Other HPV	1			27	28
Total		28	4	27	60

^a^single and mixed infections are included

^b^HPV16/18 mixed infections are counted among HPV16-positives

Among the 60 women with a CIN2+ diagnosis and a cobas 6800 valid result, the HPV test was positive in all but one sample (diagnosed as CIN2 and 4800-positive with a 39,7 Ct value). Of the 10 discordant samples among the controls, 2 were 4800-positive/6800-negative and 8 were 4800-negative/6800-positive (Table 1). Reactivity in only one of the three HPV channels was recorded in all discordant specimens, with a median Ct value of 38,05 (range 37,7-38,4) for the 4800-positive/6800-negative samples, and of 36,11 (range 31,46-37,47) for the 4800-negative/6800-positive ones (Table 2, upper panel).

**Table 2:** Comparison of results between cobas 4800 and cobas 6800 assays; samples with discordant results (upper panel) and samples with partial discordant results (lower panel). Qualitative results and Cycle threshold (Ct) values of HPV-positive specimens are reported

Sample ID	Cobas 4800	Cobas 6800
	HPV channel (Ct)	HPV channel (Ct)
722	HPV HR (37,7)	HPV NEG
731	HPV HR (38,4)	HPV NEG
642^a^	HPV HR (39,7)	HPV NEG
13	HPV NEG	HPV 18 (37,47)
18	HPV NEG	HPV 16 (36,84)
297	HPV NEG	HPV HR (31,46)
426	HPV NEG	HPV 16 (35,63)
690	HPV NEG	HPV 16 (35,43)
1018	HPV NEG	HPV 16 (36,80)
1195	HPV NEG	HPV 18 (35,67)
1198	HPV NEG	HPV 18 (36,55)
170	HPV HR (22,6)	HPV HR (17,6) + HPV 16 (35,3)
273	HPV HR (26,5)	HPV HR (19,6) + HPV 16 (35,3)
353	HPV HR (22,6)	HPV HR (19,2) + HPV 18 (34,7)
357	HPV HR (25,1)	HPV HR (19,9) + HPV 18 (35,0)
459	HPV HR (24,1)	HPV HR (21,2) + HPV 18 (33,9)
907	HPV HR (22,9)	HPV HR (19,1) + HPV 18 (35,3)

^a^ID 642: sample from a woman with a CIN2 lesion

Overall, HPV type analysis of the 124 samples positive by both systems (cases plus controls) showed complete concordance in 118 (95.2%) specimens and partial concordance in the remaining 6. The results of the partially discordant specimens are detailed in Table 2, lower panel. Reactivity to a single channel was detected by the 4800 assay in all cases; this was confirmed by the 6800 assay, which additionally displayed reactivity with another HPV channel. Of note, on the 6800 platform the Ct values for the concordant results were always lower than those for the additional positivity, indicative of a higher viral load of the concordantly detected type.

Clinical sensitivity and specificity values of the cobas 6800 HPV assay were compared to those of the 4800 assay, according to the recommendations for the clinical validation of new HPV-DNA assays [5]; the score values of the 6800 for both sensitivity (98% at 0.90 threshold) and specificity (99% at 0.98 threshold) were non-inferior to those of the 4800 (*p* = 0,0157 and *p* = 0,0056, respectively).

The intra-laboratory reproducibility of the 6800 assay was evaluated on 593 samples, 178 (30%) resulted HPV-positive at first testing. Overall qualitative agreement was recorded for 587/593 (99%), with a kappa value of 0.967 (95%CI 0.942–0.985). All 6 discordant samples (positivity for HPV16 in 2 and for other HPV in 4; median Ct values 34) were negative on the second testing. Among the samples HPV-positive in both runs, type-specific agreement was observed in 169/172 (98.3%) (Table 3).

**Table 3:** Intra-laboratory reproducibility analysis by the cobas 6800 HPV assay. Overall (i.e., hrHPV positive/negative) and type-specific HPV test agreement were 99% (587/593 samples) and 98.3% (169/172 samples), respectively. Kappa value = 0.967 (95%CI 0.942–0.985)

Cobas 6800 HPV test results [1]	Cobas 6800 HPV test results [2]				Total
		Positive			
	Negative	HPV16^a^	HPV18^b^	Other HPV	
Negative	415				415
Positive					
HPV16^a^	2	49		1	52
HPV18^a,b^			13		13
Other HPV	4		2	107	113
Total	421	49	15	108	593

^a^single and mixed infections are included

^b^HPV16/18 mixed infections are counted among HPV16-positives

The inter-laboratory reproducibility was evaluated on 460 samples (Table 4). Overall qualitative agreement was recorded for 456/460 (99.1%), with a kappa value of 0.969 (95%CI 0.941–0.990). All discordant samples displayed single reaction to one HPV channel (1 for HPV16 and 3 for other HPV). Type-specific agreement was observed in 117/119 (98.3%) samples HPV-positive in both runs.

**Table 4:** Inter-laboratory agreement analysis by the cobas 6800 HPV assay. Overall (i.e., hrHPV positive/negative) and type-specific HPV test agreement were 99.1% (456/460 samples) and 98.3% (117/119 samples), respectively. Kappa value = 0.969 (95%CI 0.941–0.990)

Cobas 6800 HPV test results [1]	Cobas 6800 HPV test results [3]				Total
		Positive			
	Negative	HPV16^a^	HPV18^b^	Other HPV	
Negative	337			1	338
Positive					
HPV16^a^	1	29		1	31
HPV18^a,b^			7		7
Other HPV	2		1	81	84
Total	340	29	8	83	460

^a^single and mixed infections are included

^b^HPV16/18 mixed infections are counted among HPV16-positives

**Fig. 1:**
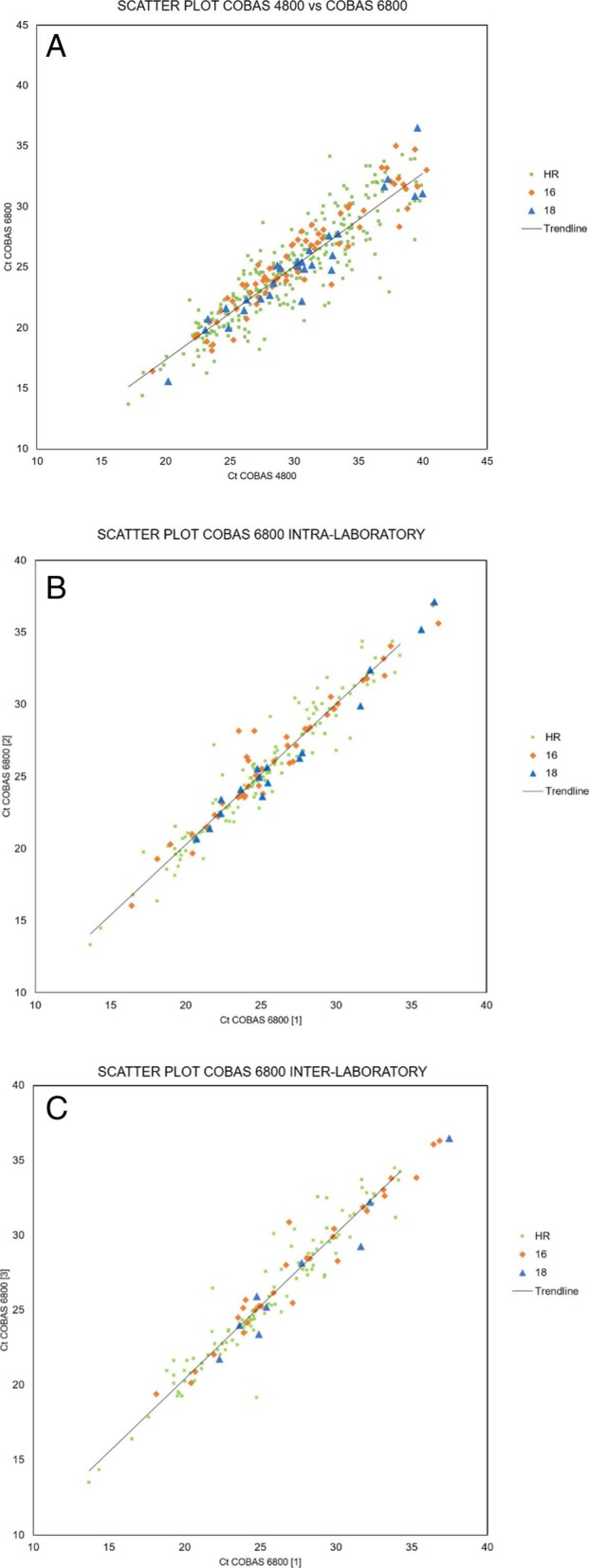
Scatter plots of the Ct values for the three HPV channels. Panel **a**: all HPV-positive samples by both systems, results on cobas 4800 vs cobas 6800; linear correlation, R^2^ = 0,78. Panel **b**: cobas 6800 intra-laboratory reproducibility; linear correlation, R^2^ = 0,92. Panel **c**: cobas 6800 inter-laboratory reproducibility; linear correlation, R^2^ = 0,92. Within each panel, the trendlines for the three channels overlapped. Ct = cycle threshold

Scatter plots of the Ct values of the HPV-positive samples are reported in Fig. 1; a linear correlation was found for all the data; coefficients for cobas 6800 intra- and interlaboratory data were higher (R^2^ = 0,92) than those for 4800 vs 6800 comparison (R^2^ = 0,78).

In each plate, two control samples were included. The 10 (8 HPV-positive, 2 HPV-negative) internal quality controls (IQC), each tested 2–6 times, were always concordant but twice for the same HPV18-positive sample that on two-out-of-three repetitions was judged as negative; additional in-depth evaluation of its analytical data disclosed that for one of the two negative results the Ct for the HPV18 channel was very high and that the mean fluorescence intensity was very weak. The 5 (4 HPV-positive, 1 HPV-negative) external quality controls (EQC) were concordant in 3 HPV-positive cases and invalid in the other 2 (one due to insufficient material, and one to a negative beta-globin result). The 2 samples resulted invalid on two different runs with the 4800 assay, both gave a valid result on the 6800 assay.

Comparison of the workflow by the two systems has shown higher laboratory operational performances of the 6800 over the 4800, and of the p 480 v2 in relation to the p 480 v1, respectively. The most important differences are summarized in Table 5. Of note, the 6800 unit contains an on-board refrigerator for reagent storage and additional storage space for consumables, thus improving both loading and long-term use of reagents and consumables. An additional improvement of the 6800 system is the automatic reporting of the Ct values for the HPV results and the internal control for the samples and the kit positive control on the assay report; the Ct values for the three HPV channels of the kit control were more uniform (median ~ 35 for all) than what recording with the 4800 system (median ranges comprised from ~ 37 to ~ 39). Finally, taking into account both the preparation and the analysis of the specimens, the amount of liquid and solid waste produced is sensibly reduced on the p 480 v2/6800 platform in comparison to the p 480 v1/4800 one (Table 5), as a result of important technological differences in all the steps. In particular, among others, while recapping by the p 480 v1 is done with new caps, the original caps are used by the p 480 v2, and while 11 tips/sample are used by the 4800, a unique tip/sample is used by the 6800.

**Table 5:** Comparison of workflow efficiencies and throughput on the cobas 4800 and cobas 6800 systems (including p 480 instruments)

	p 480 v1 + 4800	p 480 v2 + 6800
Preanalytical step:		
- samples’ aliquoting	performed by × 480	performed by p 480
Analytical steps:		
- instrument loading (reagents)	single-use vials with pouring (10 min)	re-usable reagent cartridges, no preparation needed
- reagent on-board stability	discarded after run	90 days
- user interaction	vials loading/unloading at each run	cartridge loading - unloading when empty/expired
- instrument loading (samples)	94 uncapped primary vials	up to 280 secondary tubes
- throughput (per 8 h)	192	384
- time to first 96 results	4.9 h	< 3.5 h
- time to each additional 96 results	160 min	90 min
- hands-on-time (8 h)	60 min	30 min
Instrumentation maintenance	daily	monthly
Amount of waste produced	~ 2.6 l of liquids	~ 1.7 l of liquids / ~ 40% less of sol

## Discussion

The cobas HPV test detects 14 high-risk HPV types, is based on real-time PCR technology, has been clinically validated [10, 11] and provides partial genotyping for HPV16 and HPV18, while detecting the other 12 HPV types as a pool. More recently, it has been released for use on the newly launched cobas 6800 platform (already in use for other molecular assays). In this study we compared the analytical and clinical performance, as well as other system features, of the cobas 4800 and 6800 platforms for HPV testing in cervical cancer screening, according to the Meijer’s criteria [5]. Compared to the cobas 4800 assay, the results by cobas 6800 showed an agreement > 98% for overall qualitative results between the two systems, a non-inferior clinical sensitivity and specificity, and excellent intra- and inter-laboratory reproducibility. These figures are in line with the results previously obtained in the studies conducted for the clinical validation of the cobas 4800 assay [10, 11]. The Ct values (that are inversely proportional to the viral load) obtained on the 6800 system have always been lower than those recorded by the 4800, most probably due to the different amount of target sequences analyzed by the two instruments (i.e., 25/150 μl by 4800 vs 25/50 μl by the 6800). Overall, the minor differences that emerged at the analytical level (i.e., Ct values) had no major impact on the clinical performance. Differences in semi-quantitative results in this setting are relevant only if they actually determine a change in the positive/negative result, i.e., if the value passes the threshold.

Comparison of HPV type distribution among the samples positive by both systems, and on 6800 reproducibility assays, showed a very high complete concordance for type(s) assignment (95.2% in 4800/6800 comparison, and 98.3% in both 6800 intra- and inter-reproducibility assays), in line with previous studies [10, 11, 13].

HPV16 (and to a lesser extent HPV18) has been shown to have a stronger association than other high-risk types for CIN2+ and invasive cancer [14]; as a consequence, partial genotyping for the management of HPV-positive women in the screening programs has recently been proposed as a triage test in the Australian [15] and Dutch [16] protocols, although its clinical value is still a matter of debate [17, 18]. In our study, HPV16 (either alone or mixed with other types) was detected in 19,4% (13/67) and in 26% (19/73) of the specimens by cobas 4800 and 6800, respectively, while complete type concordance was observed among the women with a diagnosis of CIN2+.

According to the European and national guidelines, HPV testing for cervical cancer screening must be centralized in large reference laboratories and performed by using clinically validated assays run on automated systems. As a consequence, the degree of automation for all the steps of the process (from sample preparation, starting from the original sample vial, to validation of the results) and the laboratory productivity are essential components to assure high-quality results and high throughput. In our study, we have shown that the workflow and several instrumentation features are sensibly improved on the new platform, as already shown also for other assays [19]. Among others, it is a compact unit comprising the thermal cycler for the real-time PCR (which implies direct transfer of the reaction plate without operator interventions); reagents and consumables are provided in a cartridge format (that avoid loading/unloading operations at each plate run); it is provided by an on-board refrigerated storing compartment for reagents (allowing their subsequent use); it can accommodate a larger number of samples (up to 280); it has shorter turn-around time for assay results and hands-on time, and is user-friendly.

## Conclusions

Our data on the HPV assay performed in a cervical screening context on the new cobas platform composed by the p 480 v2 plus the 6800 instruments, have shown high consistency of results with the cobas 4800 system in use and high intra- and inter-reproducibility, together with higher performance in workflow and laboratory productivity, thus demonstrating its suitability for use in large centralized laboratories included within population-based cervical cancer screening programs.

## Abbreviations

ASC-US: Atypical Squamous Cells of Undetermined Significance
CE/IVD: Conformité Européenne/in vitro diagnostics
CI: Confidence Intervals
CIN: Cervical Intraepithelial Neoplasia
Ct: Cycle threshold
EQC: External quality control
HPV: Human papillomavirus
IARC: International Agency for Research on Cancer
IQC: Internal quality control
PCR: Polymerase chain reaction
